# Understanding Resolvin Signaling Pathways to Improve Oral Health

**DOI:** 10.3390/ijms14035501

**Published:** 2013-03-08

**Authors:** David Keinan, Noel J. Leigh, Joel W. Nelson, Laura De Oleo, Olga J. Baker

**Affiliations:** 1 Department of Periodontics and Endodontics, School of Dental Medicine, University at Buffalo, the State University of New York, Buffalo, NY 14214-3932, USA; E-Mail: davidkei@buffalo.edu; 2 Department of Oral Biology, School of Dental Medicine, University at Buffalo, the State University of New York, Buffalo, NY 14214-3932, USA; E-Mails: njleigh@buffalo.edu (N.J.L.); joelnels@buffalo.edu (J.W.N.); lauradeo@buffalo.edu (L.D.O.)

**Keywords:** oral, resolvin, signaling pathways, inflammation

## Abstract

The discovery of resolvins has been a major breakthrough for understanding the processes involved in resolution of inflammation. Resolvins belong to a family of novel lipid mediators that possess dual anti-inflammatory and pro-resolution actions. Specifically, they protect healthy tissue during immune-inflammatory responses to infection or injury, thereby aiding inflammation resolution and promoting tissue healing. One of the major concerns in modern medicine is the management and treatment of oral diseases, as they are related to systemic outcomes impacting the quality of life of many patients. This review summarizes known signaling pathways utilized by resolvins to regulate inflammatory responses associated with the oral cavity.

## 1. Introduction

It is well established that short-lived mediators derived from arachidonic acid (AA) regulate various events related to innate-immunity, coagulation, inflammation and uncontrolled cell growth [1–13]. Fatty acid precursors are transformed into potent bioactive mediators, eicosanoids, which play a role during inflammation and its resolution [14–17]. The main bioactive products derived from AA can be inflammatory mediators, such as prostaglandins (PG) and leukotrienes (LT) or anti-inflammatory mediators, such as lipoxins (LX) [18,19]. Yet, AA is not the only fatty acid precursor involved in inflammation and its resolution. The omega-3 (ω-3) fatty acids; docosahexaenoic acid (DHA) and eicosapentaenoic acid (EPA), have also been recognized as endogenous anti-inflammatory lipid mediators that have proven to be beneficial in the treatment of many diseases [20–23]. Previous studies have demonstrated that human and animal cells convert ω-3 polyunsaturated fatty acids (PUFAs) into resolvins [24–26]. These lipid mediators were originally identified by Serhan and colleagues in a self-limited model of acute inflammation and have provided new insights indicating that resolution of inflammation is an active process [24,27].

Resolvins (resolution-phase interaction products) are short-lived autacoids, belonging to a novel family of aspirin-triggered (AT) bioactive lipids, which are synthesized during the resolution of inflammation [24,27]. They exhibit both anti-inflammatory and pro-resolving actions demonstrating the protective effects of ω-3 fatty acids [28–30]. Resolvin subtypes include the E series (RvE1-3, derived from EPA), the D series (RvD1 and RvD2, derived from DHA) and AT forms (AT-RvD1-6) [31–34]. DHA and EPA are initially converted to 17*S*-hydroperoxy-products and 18*R*-HEPE, respectively, and then are further enzymatically transformed into resolvins [24,35,36]. In the presence of aspirin, DHA is converted to a 17*R*-hydroperoxy-product that is transformed into the AT forms. The resolvins AT epimeric forms are more chemically stable than the endogenous forms, and their molecular structure has been previously elucidated [26,37].

The role of resolving lipid mediators has provided evidence that resolution of inflammation is an active programmed response enabling tissues to return to homeostasis [14–17,24,27,38]. Accordingly, resolving lipid mediators operate by counterregulating inflammation and allowing tissue repair. Here we compile studies on signaling pathways triggered by activation of resolvin receptors in systems related to the oral cavity. Understanding how resolvins signal during the resolution of inflammation might help to identify the potential advantages and limitations for their use in treating oral diseases.

## 2. Inflammation and Polyunsaturated Fatty Acids

Acute inflammation is a physiological mechanism that protects the host against local injury [39]. Under normal physiological conditions, the inflammatory response is cleared to a non-inflammatory stage, leading to restoration of normal tissue architecture and function [40–44]. Defects in these clearance mechanisms appear to be associated with persistent tissue inflammation and autoimmunity to cellular contents [35,45–47]. Tissue damage resulting from uncontrolled acute inflammatory responses causes discomfort and severely compromises normal tissue function [37].

The two main PUFAs family members, ω-3 and ω-6, are essential components of the phospholipid membrane. ω-3 must be obtained from specific foods, such as fish oil, walnuts and green leafy vegetables, while ω-6 originates from a diet rich in grains and vegetable oils [48–51]. Orally ingested EPA and DHA are rapidly mobilized to sites of inflammation during the formation of tissue exudates [52]. The essential role of ω-3 PUFAs and its mediators, EPA and DHA, in counterregulating inflammatory responses and preventing disease have been widely studied [21,23–25,31,36,38,53–56]. An ω-3 PUFA-rich diet was also found to decrease the level of pro-inflammatory cytokines in blood [57,58]. In contrast, an ω-6 PUFA-rich diet was found to significantly increase the expression levels of pro-inflammatory cytokines and decrease expression levels of anti-inflammatory cytokines [59]. Moreover, a diet with a decreased ratio of ω-3 to ω-6 PUFAs has been associated with increased incidence of inflammatory diseases [48,60–63].

Desaturation and elongation of linoleic acid (LA), an ω-6 PUFA, leads to the formation of AA in many cell types [48]. The biosynthesis of AA from LA and EPA from α-LA is mediated by the same enzyme (desaturase); this means that a high dietary intake of LA may interfere with the conversion of α-LA to EPA. AA generates inflammatory mediator-like compounds, such as PG and LT [18], and can negatively regulate the conversion of EPA from ω-3 PUFA [64]. The role of PG and LT in inflammatory responses has been widely investigated [65–71]. The LT are formed from AA by lipoxygenases (LOX) and have an important role in the mediation of chemotaxis [71], asthma [72] and inflammatory bowel disease [73].

In both acute and chronic models of inflammation, endogenous resolvins have been shown to accelerate resolution of inflammation [54,74,75]. Resolvins enhance and restore tissue integrity, since they possess both anti-inflammatory and pro-resolution mechanisms [76]. Resolvins are produced in resolving exudates *in vivo* as a byproduct of transcellular biosynthesis with human leukocytes, endothelial or epithelial cells [24]. The first step in resolvin biosynthesis involves the release of ω-3 PUFA from membrane phospholipids by phospholipase A_2_, which has been demonstrated to be responsible for DHA and EPA release in neural and retinal-pigmented cells [25,26,55,77–80]. However, in acute inflammation, it was demonstrated that DHA from peripheral blood enters the inflammatory exudate as a free fatty acid that is converted by resolving exudates to resolvins and protectins [52]. The second step during resolvin synthesis is the transformation of DHA to 17*S*-HpDHA by enzymatic conversion through (12- or 15-LOX forms) [33,37,81]. The final step, involves the capturing of 17*S*-HpDHA by polymorphonuclear neutrophils (PMNs) and transformation into RvD isoforms by the different LOX isoenzymes [33,37,81]. In the presence of aspirin, 12- or 15-LOX activities in epithelial cells are transformed into cyclooxygenases by COX-2 activation [37]. This leads to the transformation of EPA to 18*R*-HEPE and 17*R*-HpDHA to AT-RvD1 by 5-LOX [37].

## 3. Signaling Pathways

**Figure 1:**
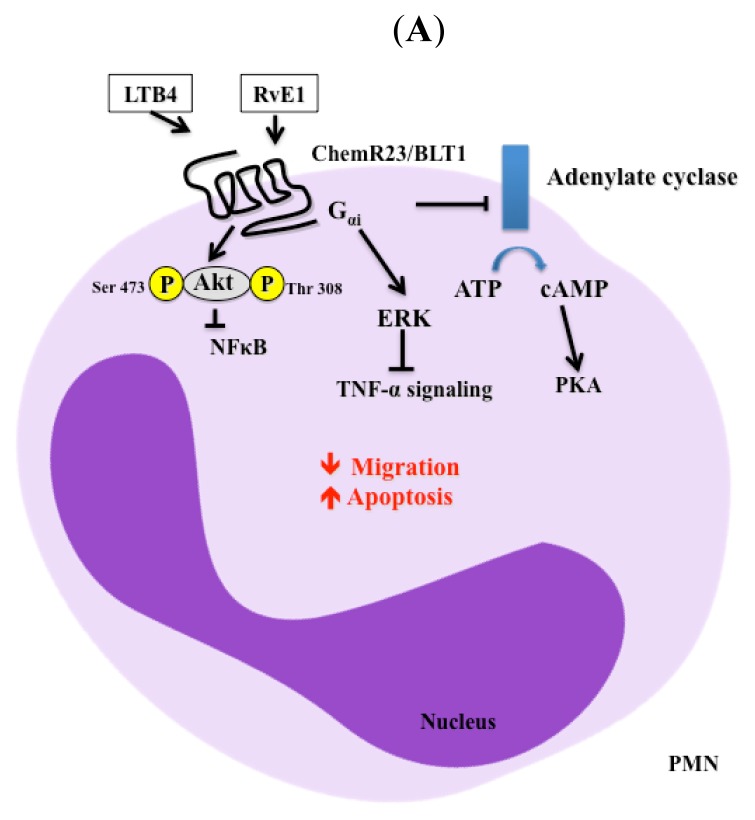

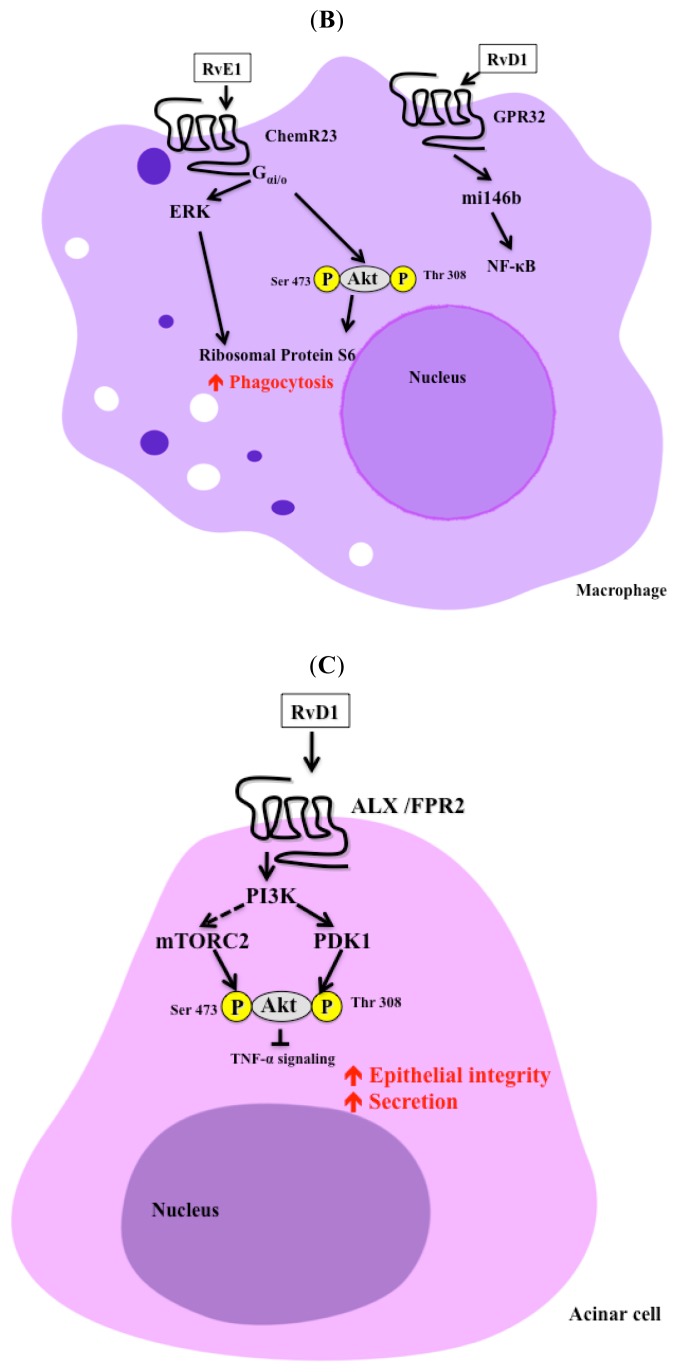
Resolvin signaling pathways in different cell types. (**A**) In polymorphonuclear neutrophils (PMNs), RvE1 binds to ChemR23, activates G_αi/o_, which activates extracellular signal-regulated kinase (ERK), and eventually blocks TNF-α signaling. Alternatively, RvE1 binds to BLT1 and blocks the activation of adenylate cyclase and NFκB signaling; (**B**) In Macrophages, RvE1 binds to the ChemR23 receptor and activates Akt via mTOR and alternatively blocks TNF-α signaling via ERK. Also, RvD1 binds to GPR32 to enhance miRNA expression and activate transcription factors leading to increased phagocytosis; (**C**) In acinar cells, RvD1 binds to the ALX/FPR2 receptor leading to Akt activation and blocking TNF-α signaling.

The signaling cascades for resolvins trigger different transcription factors depending on the specific resolvin molecule and its receptor. RvE1 selectively interacts with two distinct G protein coupled receptors (GPCRs) on various cell types to promote resolution of inflammation. Binding of RvE1 to the ChemR23 receptor phosphorylates extracellular signal-regulated kinase (ERK) via G_αi/o_ activation. This signaling mechanism attenuates TNF-α-mediated NF-κB activation in PMNs from humans and mice [82]. The ChemR23 receptor is expressed abundantly in macrophages and dendritic cells [83]. Additionally, it can be found in human endothelial cells [84], kidney [85], lung [86] and salivary glands [87]. Alternatively, RvE1 can cause both ligand and receptor-dependent phosphorylation of Akt in ChemR23-transfected Chinese hamster ovary cells [88]. In the system described above, the ribosomal protein S6 served as a downstream target of the phosphatidylinositol 3-kinase PI3K/Akt signaling pathway, as well as the Raf/ERK pathway [88]. RvE1 also enhanced phagocytosis of zymosan A by human macrophages, which were inhibited by PD98059 (MEK-1 inhibitor) and rapamycin (mTOR inhibitor) [88]. These studies indicate that RvE1 initiates direct activation of ChemR23 and signals receptor-dependent phosphorylation. RvE1 also binds to the LTB4 receptor 1 (BLT1) on human PMNs inhibiting adenylate cyclase [82]. These studies demonstrate the ability of RvE1 to selectively bind to GPCRs and activate intracellular signaling in different cell types (Figure 1A,B). Furthermore, RvE1 decreased LTB4-induced NF-κB activation and blocked PMN chemotaxis and infiltration [82]. RvE1 has also been shown to enhance cell migration in corneal epithelial cells at levels comparable to those induced by epidermal growth factor (EGF). These increases were associated with phosphorylation of the EGF receptor (EGFR) and downstream signaling involving PI3K and p38. Interestingly, cleavage of EGF by metalloproteases suppressed RvE1-induced stimulation of EGFR/PI3K/Akt phosphorylation and cell migration. These results indicate that RvE1 is likely to cause transactivation of EGFR [89]. Recently, another member of the RvE family, RvE2, was found to partially share the BLT1 receptor with RvE1 at a potency similar to that of RvE1 and is essentially equipotent with RvE1 in limiting PMN infiltration. However, RvE2 was found to be a weak activator of the ChemR23 receptor [90]. These results may explain the finding that RvE1 is more potent than RvE2 in various *in vivo* systems depending on the cell type and tissue. RvE2 also enhanced phagocytosis and interleukin (IL)-10 production, suggesting that these RvE2 actions may be transduced by additional receptors that have yet to be discovered.

The RvD family shares similar signaling mechanisms as the RvE family, as they also activate GPCRs. Specifically, RvD1 activity is mediated by two GPCRs termed ALX/FPR2 and GPR32 [81,91]. The ALX/FPR2 has been shown to bind lipid and protein ligands, eliciting either pro-inflammatory or anti-inflammatory responses. GPR32 is an orphan receptor that uses a β-arrestin-based ligand receptor system that elicits inflammatory and pro-resolvin responses. Both RvD1 and its 17(*R*) epimer reduce PMN transendothelial migration and exhibit a dose-dependent reduction in leukocyte infiltration in a murine model of peritonitis [24]. In glial cells, RvD1 blocks upregulation of TNF-α-induced IL-1β transcript (a marker of neural injury) [25]. In salivary glands, RvD1 also blocks TNF-α signaling through Akt activation leading to enhanced tissue integrity and cell polarity (Figure 1C) [92]. These studies demonstrate that RvD1 is able to block intracellular signaling pathways related to inflammatory processes in a variety of cell types.

Both RvD1, as well as RvE1, blocked cell mucous secretion mediated by CysLT_1_ receptors in cultured rat goblet cells [93]. Specifically, RvD1 and RvE1 prevented increases in intracellular calcium concentration mediated by ERK activation through LTD4 [93]. These studies indicate that RvD1 and RvE1 trigger signaling mechanisms that modulate resolution of inflammation in the eye.

Recently, several microRNAs (miRNA) were identified in the resolution phase of murine peritonitis [94]. In this *in vivo* experimental system, RvD1 up-regulated miR-21, miR-146b and miR-219 and downregulated miR-208a [94]. RvD1-miRNAs identified here were able to target cytokines and proteins involved in the immune system, for instance, miR-146b targeted NF-κB signaling [94]. Additionally, miR-219 targeted 5-LOX and consequently reduced LT production [94]. Taken together, these results establish a novel resolution circuit involving RvD1 receptor-dependent signaling of specific miRs [94]. A later study demonstrated RvD1 is highly selective for pro-resolving agonists of hALX/FPR2 and hGPR32 [91]. RvD1 upregulated miR-208a, a miRNA that targets programmed cell death protein 4 (a signaling molecule that up-regulates IL-10 in human macrophages) [91]. In summary, the studies cited above demonstrate the selectivity of RvD1 interactions with receptors ALX/FPR2 and GPR32 in modulating miRNAs during the resolution of inflammation.

## 4. Resolvins and Immune System

The ω-3 PUFAs are appreciated for their beneficial actions in the immune system [95], for instance, the presence of DHA, EPA and their mediators are found at local sites of inflammation [35,96–100]. During acute inflammation, PMN produce oxygen radicals and release hydrolytic and proteolytic enzymes [101–103]. These byproducts are capable of killing bacteria and need to be removed from the site of inflammation. Therefore, failure of this mechanism might cause tissue damage and chronic inflammation. Apoptosis of PMN is a physiological process for removal of PMN from inflammatory sites by opsonization and recognition by macrophages [104–106]. Abolition of inflammation is also mediated by secretion of anti-inflammatory cytokines, such as IL-10 and TGF-β [107]. However, when there is a failure to resolve acute inflammation, there is necrosis of PMN. This may rupture cell membrane, release of intracellular content and cause tissue damage. The progress of these events results in chronic inflammation that includes abscess formation, scarring and autoimmunity.

Resolvins regulate the immune system by controlling functions of specific cell types. For instance, RvD1 differentially modulates primary human macrophage responses to lipopolysaccharides, depending on the context in which this molecule is presented to the macrophage [108]. Resolvins and protectins have been shown to stimulate innate killing mechanisms to manage bacterial loads and stimulate clearance of bacteria [31]. RvE1 is a potent inhibitor of leukocyte infiltration, dendritic cell migration, IL-12 production and PMN transendothelial migration [26,109]. Furthermore, RvE1 was found to negatively regulate the development of an allergic inflammation *in vivo*[110]. Other studies demonstrated that RvE2 stimulates host-protective actions throughout initiation and resolution of the innate immune responses [90]. Additionally, RvE3 has proven to be a potent inhibitor of PMN chemotaxis *in vitro* and *in vivo*[34]. Recently, it was demonstrated that in *E. coli* infections, the combination of RvD1, RvD5 and protectin D1 (a dihydroxy product formed in inflammatory exudates), together with antibiotics, increased antimicrobial responses in mouse peritoneum [111]. The studies stated above indicate that resolvins block excessive inflammatory responses and promote resolution of inflammation as follows: (a) blocking cytokine production; (b) reducing PMN transendothelial migration and (c) increasing macrophage activity resulting in the clearance of apoptotic cells and debris from inflamed areas.

## 5. Resolvins and Pain

The precursor of resolvin D series, 17*S*-HpDHA, modulates both the genesis and the maintenance of mechanical hyperalgesia in an arthritis model in rats [112]. This anti-hyperalgesic effect in acute inflammation seems likely to be mediated by inhibition of both NF-κB and COX-2 in the peripheral nervous system [112]. These effects were partly related to decreased production of TNF-α and IL-1β in rat hind paw [112]. RvE1 can reduce neuropathic pain by several mechanisms, which include inhibition of the following: (a) TNF-α synthesis release and downstream signaling [113]; (b) transient receptor potential ion channel signaling [114] and (c) peripheral inflammation via enhancing the phagocytic activity of macrophages [113]. Furthermore, treatment with RvE1, three weeks after nerve injury, transiently reduced mechanical allodynia and heat hyperalgesia [115]. The studies listed above suggest that resolvins could be used as a novel class of analgesics to treat inflammatory pain. Two advantages over current drug therapies to treat pain include; high potency and endogenous production in the body.

## 6. Resolvins and Coagulation

Blood clotting is an important mechanism to help the body repair injured blood vessels. This mechanism involves several steps, including: (a) constriction of blood vessels; (b) platelet aggregation and (c) stabilization of the blood clot [116–119]. These processes are mediated primarily by activation of thromboxane and PG. In human platelet-rich plasma, RvE1 selectively blocked both adenosine diphosphate (ADP)-stimulated and thromboxane-stimulated platelet aggregation in a concentration-dependent manner [120]. Another study demonstrated that RvE1 possesses regulatory actions, such as reduction of ADP-stimulated P-selectin surface mobilization and actin polymerization [121]. The specific platelet actions of RvE1 selectively engaged with ADP activated platelets may contribute to both resolution of vascular inflammation and ADP-dependent platelet activation [121]. RvE2 may also contribute to homeostasis, as it rapidly downregulates surface expression of human leukocyte integrins in whole blood. Additionally, it dampens responses to platelet-activating factor, a potent activator of platelets and leukocytes [90]. Together, these results indicate that RvE1 selectively regulates platelets, which are critical cell components for blood coagulation.

## 7. Resolvins and Periodontitis

Periodontitis is a chronic inflammatory disease caused by the release of immune mediators, resulting in destruction of the alveolar bone and periodontal connective tissue [122]. The process of bone resorption is a result of proteolysis and acid production mediated by osteoclasts [123]. Additionally, in this process, there is a massive expression of vacuolar-type H^+^-ATPase that enables bone degradation [124].

The mechanism by which bone resorption is regulated involves different factors, including PGE_2_, which activates osteoclasts [125] while influencing their number and function [126]. In contrast, RvE1 was found to inhibit osteoclast growth and bone resorption by interfering with its differentiation [127]. A previous study indicated that topical application of RvE1 to rabbit periodontal tissue conferred dramatic protection against tissue and bone loss associated with periodontitis [75]. In that study, it was also demonstrated that PMNs from localized aggressive periodontitis were refractory to resolving molecules of the lipoxin series. However, PMNs responded to RvE1, which stopped superoxide anion generation by binding at a site that is functionally distinct from the aspirin-triggered lipoxin receptor [75]. These studies revealed the potential of using resolvins for prevention and treatment of periodontal disease. Furthermore, they provide a new role for resolvin signaling in the pathogenesis of periodontal disease.

## 8. Resolvins and Salivary Gland Function

Sjögren’s Syndrome (SS) is an autoimmune disease characterized by xerostomia (dry mouth) and Keratoconjunctivitis sicca (dry eyes) [128]. Such symptoms are clinically detectable only after salivary and lacrimal glands display chronic inflammation, a point at which current therapies have no benefit [129–131]. RvD1 receptor activation promotes resolution of inflammation and tissue repair in salivary epithelium, which may have relevance in the restoration of salivary gland dysfunction associated with SS [92]. It was demonstrated that RvD1 treatment in Par-C10 cells prevents TNF-α mediated disruption of salivary epithelial formation. Also, RvD1 enhanced cell migration and cell polarity via PI3K/Akt signaling in Par-C10 cells [92]. These studies indicate that activation of ALXR/FPR2 with RvD1 could be used not only to block inflammation, but also to improve tissue repair and regeneration in damaged salivary glands.

## 9. Conclusions

**Figure 2:**
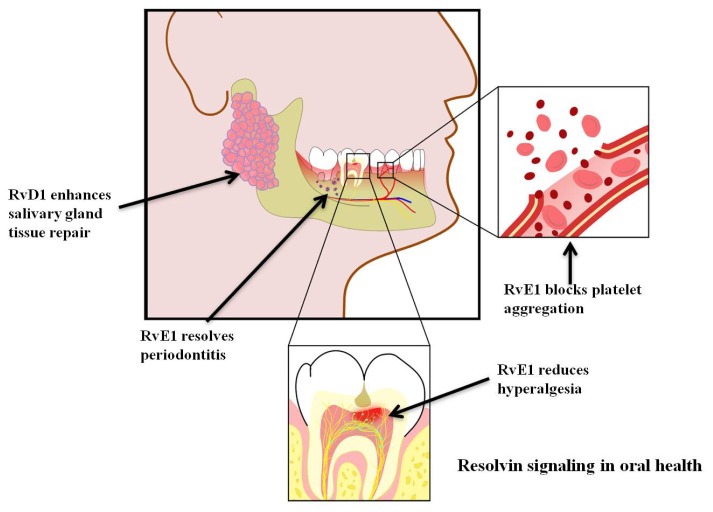
The potential uses of resolvins to improve oral health. Resolvins have been found to improve salivary gland epithelial integrity, resolve inflammation in periodontitis, reduce hyperalgesia and decrease platelet aggregation in several *in vivo* and *in vitro* models.

Here we highlighted the signaling mechanisms of resolvins in different systems. Further understanding of these mechanisms may provide clues to develop new therapies for oral diseases. Resolvins enhance and restore tissue integrity, since they possess both anti-inflammatory and pro-resolution properties. This review is relevant for the dental field given the multiple inflammatory conditions commonly observed in the oral cavity (Figure 2). In an ideal clinical setting, we would be able to control pain, inflammation, infection, autoimmunity and bleeding defects by modulating resolvin signaling pathways.
